# 

**Published:** 1873-10

**Authors:** 


### CONTENTS.

**CONTRIBUTIONS.**

PAGE.

Enjoying Poor Health, BEN H. PRATT, A. M ........... 165
Certain Powers and Properties of Water. P. H. HAYES, M. D........ 167

**CORRESPONDENCE.**

Letters and Answers........ 176

**CLIPPINGS.**

Sick Headache........ 169

**EDITORIAL.**

PAGE

Medical Items and News........ 170
Household Hints and Recipes........ 179
Elmira Surgical Institute........ 182
Spinal Curvature........ 182
Purulent Ophthalmia........ 185
Club Foot........ 187
Chit-Chat........ 189
Books and Periodicals........ 191

